# Comparison of toric intraocular lens axis measurement by slit lamp versus photography

**DOI:** 10.1007/s00417-026-07115-5

**Published:** 2026-02-10

**Authors:** Eshchar Raviv Sasportas, Adi Levy, Ehud Assia, Guy Kleinmann

**Affiliations:** 1 Department of Ophthalmology, Edith Wolfson Medical Center, Holon, Israel; 2 Gray Faculty of Medical and Health Sciences, Tel Aviv University, POB 39040, Tel Aviv , 69978 Israel; 3 Ein-Tal Eye Center, Tel Aviv, Israel; 4 Goldschleger Eye Institute, Sheba Medical Center, Ramat Gan, Israel

**Keywords:** Toric intraocular lens, Axis misalignment, Slit lamp, Photography, Astigmatism, Cataract surgery

## Abstract

**Purpose:**

To investigate the accuracy of slit lamp measurement of toric Intraocular lens (IOL) axis compared with photography and software measurement of the same IOL.

**Methods:**

The medical charts of 102 eyes of 86 patients who underwent cataract surgery with toric IOL implantation in Ein-Tal eye center, Israel, were retrospectively reviewed. Preoperative data includes demographics, biometry, and calculated IOL. Post-operative data includes visual acuity, refraction, and the IOL axis as measured at the slit lamp and using the Goniotrans eye photograph software. The difference between the two measurements was calculated.

**Results:**

The mean absolute difference between the slit lamp and the photography measurement was 2.13 ± 1.9^o^ (range 0.0-9.0^o^). In the against the rule (ATR) cases (*n* = 50) 48% were counterclockwise (CCW), 32% were clockwise (CW) and in 20% there was no difference, the average absolute deviation was 0.4 ± 1.8^o^ CCW, range 0.5-7.0^o^. In the with the rule (WTR) cases (*n* = 40), 52% were CCW, 23% were CW and in 25% there was no difference, the average absolute deviation was 1.0 ± 2.0^o^ CCW, range 0.5-9.0^o^. In the oblique cases (*n* = 12) 58% were CW, 25% were CCW and in 17% there was no difference, the average absolute deviation was 0.37 ± 1.9 ^o^ CW, range 0.5-5.5^o^.

**Conclusions:**

Average discrepancy between the slit lamp and photography measurement was small, but in an individual case it was found to be as high as 9.0^o^. It can be significant when rotation of toric IOL is needed. When photography is not available slit lamp measurements should be used as is.

## Key messages

*****What is known***:**

- Accurate alignment of toric intraocular lenses is crucial for optimal visual outcomes, as postoperative misalignment significantly reduces their effectiveness.

*****What is New***:**

- Although average differences between standard slit lamp vs. software-assisted photographic measurements of toric IOL axis are small, individual discrepancies can reach up to 9°.
- Software-based photographic measurement is recommended for precise planning of toric IOL repositioning to address potential outliers in slit lamp assessment.

## Introduction

Up to 40% of the patients undergoing cataract surgery present with visually significant corneal astigmatism [1, 2]. To address this, toric intraocular lenses (IOLs) have proven to be effective in reducing corneal astigmatism during cataract surgery [3]. Toric IOLs are widely recognized as the most reliable and predictable method for correcting corneal astigmatism, offering patients improved uncorrected visual outcome [4] and are used in about 20% of cataract cases [5, 6]. The presence of noticeable residual astigmatism following the implantation of a toric IOL can lead to dissatisfaction for both the patient and the surgeon [7]. 

Residual refractive astigmatism after toric IOL implantation arises from an incorrect amount of cylinder power or misalignment of the lens. Every degree of misalignment of the toric IOL from the intended axis results in a 3.3% decrease in the toric power correction. One of the primary reasons for toric misalignment is post-operative rotation, taking place in 3% of the cases [8], which commonly takes place shortly after the surgical procedure [9, 10]. Accurate postoperative measurement of the toric IOL axis is critical for evaluating the success of the astigmatism correction, maximizing the visual outcomes and the back calculations.

The purpose of our study was to investigate the accuracy of the common practice slit lamp measurement of the toric IOL axis, with the gold standard software-based axis measurements of the photograph of the toric IOLs.

## Methods

**Fig. 1:**
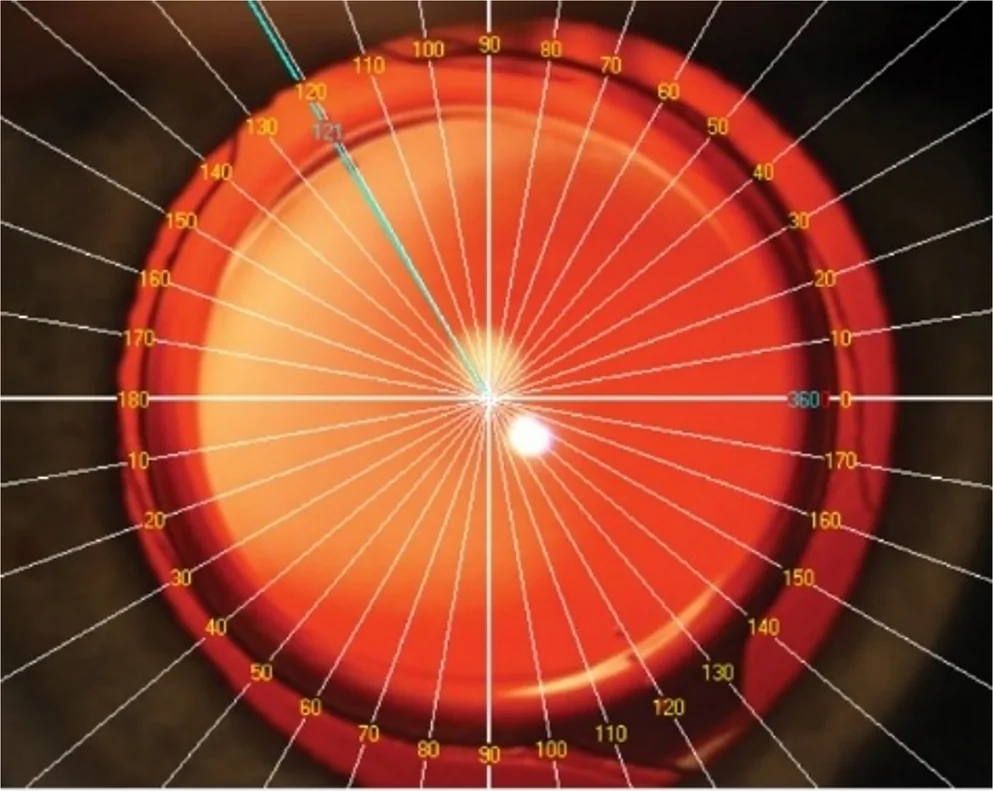
Goniotrans eye photography

A retrospective chart review of patients who underwent cataract surgery with toric IOL implantation by two experienced surgeons (EIA, GK) between 2013 and 2022 at the Ein Tal Eye Center, Tel Aviv, Israel. Ethics approval for this study was obtained from the IRB Committee of the Edith Wolfson Medical Center. Included in the study were eyes that had full preoperative data including demographics, biometry, and calculated IOL parameters, uncomplicated surgery, and postoperative follow-up that completed at least 3 weeks follow-up including uncorrected and best corrected visual acuity, refraction, IOL axis measurement obtained by an experienced certified OD and by photography using the Goniotrans eye photograph software (Eventos Médicos y Sociales, SL). We excluded eyes that did not meet all of our inclusion criteria. The Goniotrans photography method is based on a virtual angle conveyor, annotating the angular position of ocular structures based on the image of the eye (Fig. 1).

The mean absolute difference between the slit lamp and photography measurements was calculated to evaluate the disparity between the two measurement methods. Further analysis was performed according to the IOL orientation: with the rule (WTR), against the rule (ATR), and oblique astigmatism. Corneal astigmatism was classified as WTR when the steepest meridian was within the range of 60° and 120°, and ATR when it fell between 0° and 30° or 150° and 180°. All other cases, where the steepest meridian was greater than 30° and less than 60°, or greater than 120° and less than 150°, were categorized as oblique astigmatism. Clockwise (CW) and counterclockwise (CCW) designations were used to indicate the direction of the difference between the slit lamp and photography measurements.

The statistical analysis utilized Excel software (version 2401; Microsoft Office 365 MSO). We computed the weighted average for each deviation type (ATR, WTR, Oblique) and direction (CW, CCW) independently, including cases of null deviation. T-tests were then applied to these results, considering a P value less than 0.05 as statistically significant. A sample size calculation was performed based on a mean absolute difference of 2.0° with a standard deviation of 2.0°, aiming to detect a significant difference of 1.0° with 90% power and a significance level of 0.05. This resulted in a required sample size of 81 eyes.

## Results

One-hundred and two eyes of 86 patients with toric IOL implantation were included in the study. The patient’s demographics, preoperative biometric data, postoperative measurements, and the IOL characteristics are summarized in Table 1.

**Table 1:** Demographics, preoperative biometric data, IOL characteristics and postoperative visual outcome

Variable	Number	Range
Age (years), mean ± SD	66.5 (10.4)	41–88
Male gender (%)	49	
Laterality, RE (%)	55	
Biometry		
Axial length (mm), mean ± SD	24.7 (1.7)	20.6–28.2
Average K, mean ± SD	43.9 (2.1)	39.4–57.0
Astigmatism (D), mean ± SD	1.8 (1.3)	0.3–8.7
IOL power (D), mean ± SD	18.1 (5.1)	7.5–33.0
Toric correction (D), mean ± SD	2.43 (1.8)	1.0–11.0
IOL type (%)		
Tecnis Symphony ZXT	47	
AcrySof SN6ATx	29	
Finevision POD FT	17	
Other*	7	
Post operative results:		
Sphere (D), mean ± SD	−0.31 (0.5)	−2.25**- (+ 0.75)
Astigmatism (D), mean ± SD	−0.06 (0.5)	−2.25***- (+ 0.75)
SE (D), mean ± SD	−0.4 (0.5)	−3.0–0.0
BCDVA (LogMAR), mean ± SD	0.07 (0.13)	−0.1 - (0.88***)
BCIVA (LogMAR), mean ± SD	0.09 (0.1)	−0.1- (0.3)
BCNVA (LogMAR), mean ± SD	0.05 (0.05)	0.0 - (0.3)

*SD* standard deviation, *SE* spherical equivalent, *BCDVA* best corrected distance visual acuity, *BCIVA* best corrected intermediate visual acuity, *BCNVA* best corrected near visual acuity

* Other include: Biflex toric IOL, Synergy DFW, Hanita VisTor

** Monovision included in this data

***included keratoconus

The total mean absolute difference between the slit lamp and photography measurement for the entire study cohort was 2.13 ± 1.9^o^ (range 0–9^o^). The analysis of the measurements was further divided into three categories, 50 eyes of against-the-rule (ATR) astigmatism, 40 eyes of with-the-rule (WTR) astigmatism, and 12 eyes of oblique astigmatism.

Among the ATR astigmatism eyes, the average absolute difference between the slit lamp and photography measurements was 0.4 ± 1.8^o^ CCW (range 0.5–7.0.5.0^o^, *P* = 0.2). 48% exhibited CCW difference (mean 2.5 ± 1.5^o^, range 0.5–7.0.5.0^o^, *P* = 3 × 10^− 8^), and 32% displayed CW difference (mean 2.53 ± 1.9^o^, range 0.5–7.0.5.0^o^, *P* = 0.12). In 20% of the ATR cases, no difference between the slit lamp and photography measurement was documented (Table 2).

**Table 2:** Slit lamp and goniotrans measurements difference in these different groups

Variable	Mean ± SD (Range)	*p*-value	*N* (% of total)
ATR (*n* = 50)			
Counter clockwise	2.5 ± 1.5 (0.5 to 7)	0.00000003	24 (48)
Clockwise	2.53 ± 1.9 (0.5 to 7)	0.12	16 (32)
No difference	0		10 (20)
Total mean absolute ATR difference	0.4 ± 1.8 (0.5 to 7) CCW	0.2	50 (100)
WTR (*n* = 40)			
Counter clockwise	3.14 ± 2.1 (0.5 to 9)	0.00001	21 (52)
Clockwise	2.89 ± 1.4 (1.0 to 5.0)	0.00017	9 (23)
No difference	0		10 (25)
Total mean Absolute ATR difference	1.0 ± 2.0 (0.5 to 9) CCW	0.03	40 (100)
Oblique (*n* = 12)			
Counter clockwise	3.5 ± 1.1 (2 to 4.5)	0.02	3 (25)
Clockwise	2.14 ± 1.4 (0.5 to 5.5)	0.01	7 (58)
No difference	0		2 (17)
Total mean absolute Oblique difference	0.37 ± 1.9 (0.5 to 5.5) Clockwise	0.33	12 (100)

Among the eyes with WTR astigmatism, the average absolute difference between the slit lamp and photography measurements was 1.0 ± 2.0^o^ CCW (range 0.5–9.0.5.0^o^, *P* = 0.03). 52% showed CCW rotation (mean 3.14 ± 2.1^o^, range 0.5–9.0.5.0^o^, *P* = 1 × 10^− 5^), and 23% displayed CW rotation (mean 2.89 ± 1.4^o^, range 1.0–5.0.0.0^o^, *P* = 1.7 × 10^− 4^). In 25% of the WTR cases, no difference was documented (Table 2).

Among the oblique astigmatism eyes, the average absolute difference between the slit lamp and photography measurements was found to be 0.37 ± 1.9^o^ CW (range 0.5–5.5.5.5^o^, *P* = 0.33). 58% exhibited CW difference (mean 2.14 ± 1.4^o^, range 0.5–5.5.5.5^o^, *P* = 0.01), 25% showed CCW difference (mean 3.5 ± 1.1^o^, range 2.0–4.5.0.5^o^, *P* = 0.02), and in 17% of the cases, no difference was documented (Table 2).

## Discussion

In this study, we found a relatively small (2.13 + 1.9^o^) mean absolute difference between the measurement of the toric IOL axis as measured using the slit lamp bio microscopy compared with a slit lamp photograph and the Goniotrans software, 3 weeks after the surgery. The range of differences was up to 9 degrees. This difference can be significant when IOL rotation is needed in the back calculation of the desired new axis and the proper realignment axis. The difference was found in all astigmatism orientations. It was not consistent in all of them, presenting clockwise and counterclockwise differences in most cases, with agreement between the 2 methods in only 20–25% of the eyes.

Toric IOL implantation is a successful way for corneal astigmatism elimination and improving uncorrected visual acuity. Accurate measurements, advanced power calculations, and proper IOL alignment are required for the success of the toric IOL. IOL misalignment can arise from various factors such as measurement error, marking technique, capsulorhexis size, IOL edge coverage, corneal incision sealing, rotational stability, and surgeon expertise. Misalignment can occur during surgery or due to postoperative rotation. Advanced modalities, image-guided, and aberrometry systems contribute to the reduction of pre- and intra-operative alignment errors [11–13]. 

Toric IOL postoperative realignment may be necessary in 0.65% to 3.3% of the eyes [14–16]. Realignment should be considered when improved uncorrected visual acuity can be expected [11–13]. Each degree of IOL misalignment results in a 3.3% loss of the effective power. Misalignment exceeding 30 degrees can introduce cylindrical power in a new axis, reducing visual acuity. A small axis rotation of up to 10 degrees can still provide satisfactory visual acuity [17]. The decision on IOL rotation is based on the patient’s uncorrected visual acuity, refraction, best-corrected visual acuity, Toric IOL power, and the measurement of the IOL axis in the eye.

Assessment of toric IOL axis is influenced by errors secondary to cyclorotation, head tilt or inadequate camera adaptation. Many techniques described for the measurement of the toric IOL axis, including slit lamp examination, digital overlay methods, vector analysis with residual refraction and keratometry values, anterior segment optical coherence tomography, and wavefront aberrometry [18–23]. Most surgeons are using the slit lamp measurement to evaluate the toric IOL axis. However, the accuracy of this method is limited by the reasons mentioned above, as well as the increments on the slit-lamp measuring scale [24]. 

Viestenz et al. investigated the rotational stability of eyes with standardized photography. They compared fundus photography taken six months apart to quantify the amount of cyclorotation and assess its impact on the measurement of the toric IOL postoperative axis. The mean absolute autorotation was 2.3°; in 36% of the eyes, there was a rotation of 3° or more, and in only 9%, there was no rotation. Their findings indicated that employing bifocal photography with a constant head position has the potential to reduce intrinsic errors in documenting the axial orientation of toric IOLs. This improvement increases the accuracy in establishing the toric IOL axis [8, 25, 26]. 

Kasthurirangan et al. assessed the rotational stability of a toric IOL utilizing custom-developed software designed to analyze slit-lamp photographs. The software uses iris and sclera landmarks to align the baseline image and later images for comparison. The software validation revealed a high precision of 0.02° when using protractor images and 2.22° when employing slit-lamp photographs. This underscores the software’s accuracy and efficiency in swiftly analyzing slit-lamp images to ascertain the degree of postoperative toric IOL rotation [27]. 

Sanders et al., quantified the measurement error caused by head tilt or cyclorotation in the measurement of rotational stability. They used high-resolution retroillumination photography to identify the toric IOL axis markings and photography of the iris and conjunctival/scleral vessels location in 37 eyes. Toric IOL rotation analysis was performed with and without iris/vessel registration correction. The mean difference was 0.07°±1.35 and the maximum difference was 4.4°. They conclude that by using this method they reduced the mean absolute toric IOL axis rotation measurement error by a factor of 4.5 [28]. 

Jiang et al. evaluated the accuracy of CASIA2 and iTrace in measuring postoperative toric IOL orientation without mydriasis. CASIA2 demonstrated significantly greater precision and narrower limits of agreement compared with the iTrace, with improved accuracy in eyes with pupil diameters equal or larger than 4 mm under semi-dark conditions [29].

The above methods are complex, requiring specialized imaging techniques and a comparison of images of the same patient over time. These sophisticated approaches, despite the accuracy and efficiency demonstrated, are not always practically feasible for routine postoperative assessments, as they consume significant time and necessitate the acquisition of specific equipment and a trained team for operation. In our research, we compared slit-lamp measurement of the toric IOL axis to a simple and easy measurement method that requires minimal resources - only a photo-slit-lamp and free available software for a quick and straightforward assessment.

Methods using a cellular phone camera for slit lamp photography and toric IOL axis measurements have also been described [30, 31]. In 2014, Teichman et al. showed a simple method for measuring the toric IOL axis with a cellular phone camera (iPhone 5 S, Apple, Inc.). They used different software to perform the image analysis; among them is the Goniotrans iPhone application we used in our study [32]. The Goniotrans software (available online for free download) is a modern and user-friendly alternative for assessing the IOL axis. The Goniotrans software utilizes a virtual protractor superimposed on the eye image to analyze the alignment of toric IOLs [33]. In cases where a photo-slit-lamp with a built-in camera is unavailable, capturing images with a mobile phone is also a viable option.

Our study strengths include a substantial sample size, providing a robust foundation. The inclusion of multiple types of lenses enhances the generalizability of the findings, reflecting the diversity encountered in clinical practice. An additional aspect is the consideration of all astigmatism orientations apart and together, offering a comprehensive analysis that captures variations across different lens orientations. The utilization of a simple and user-friendly program, Goniotrans software, ensures accuracy and repetitiveness in the assessment of toric IOL axis, promoting the feasibility of its application in routine clinical settings. The study’s limitations include its retrospective nature and reliance on data from a single center, potentially introducing selection bias and limiting external validity. Despite these limitations, the study’s strengths contribute valuable insights into the challenges and practical solutions associated with toric IOL axis measurement in cataract surgery.

The discrepancies observed in our study between slit lamp and photography measurements indicate the need for careful consideration of measurement techniques in cases of toric IOL rotation. By employing photography and software-based methods, surgeons can enhance the precision of the axis determination, which will improve the IOL back calculation and new axis determination. Nevertheless, in cases when photography and software measurements are not available, we found that the slit lamp measurement should be used as is. Further research is warranted to investigate measurement discrepancies and refine the toric IOL axis measurement techniques.

## Data Availability

The datasets analysed during the current study are available from the corresponding author on request.
